# Subcutaneous Panniculitis-like T-Cell Lymphoma: Diagnostic Challenge and Successful Multimodal Management with Integra^®^ Dermal Matrix—Case Report and Review of the Literature

**DOI:** 10.3390/diseases13070201

**Published:** 2025-06-30

**Authors:** Daniel Pit, Teodora Hoinoiu, Bogdan Hoinoiu, Simona Cerbu, Maria Iordache, Adrian Vaduva, Diana Szilagyi, Claudia Ramona Bardan, Panche Taskov, Zorin Petrisor Crainiceanu, Miruna Samfireag, Razvan Bardan

**Affiliations:** 1 Doctoral School, Victor Babes University of Medicine and Pharmacy, E. Murgu Square, No. 2, 300041 Timisoara, Romania; daniel.pit@umft.ro (D.P.); claudia.bardan@umft.ro (C.R.B.); panche.taskov@umft.ro (P.T.); 2 Discipline of Clinical Practical Skills, Department I Nursing, Faculty of Medicine, Victor Babes University of Medicine and Pharmacy, 300041 Timisoara, Romania; samfireag.miruna@umft.ro; 3 Center for Advanced Research in Cardiovascular Pathology and Hemostaseology, Victor Babes University of Medicine and Pharmacy, 300041 Timisoara, Romania; 4 Department of Oral Rehabilitation and Dental Emergencies, Faculty of Dentistry, Victor Babes University of Medicine and Pharmacy, P-ta Eftimie Murgu 2, 300041 Timisoara, Romania; 5 Interdisciplinary Research Center for Dental Medical Research, Lasers and Innovative Technologies, 300070 Timisoara, Romania; 6 Department XV of Orthopaedics, Traumatology, Urology and Medical Imaging, Discipline of Radiology and Medical Imaging, Victor Babeș University of Medicine and Pharmacy, 300041 Timișoara, Romania; cerbu.simona@umft.ro; 7 Department of Hematology, Victor Babes University of Medicine and Pharmacy, 300041 Timisoara, Romania; iordache.maria@umft.ro; 8 Multidisciplinary Research Center for Malignant Hematological Diseases (CCMHM), Victor Babes University of Medicine and Pharmacy, 300041 Timisoara, Romania; 9 Department of Pathology, Faculty of Medicine, ANAPATMOL Research Center, Victor Babes University of Medicine and Pharmacy, 300041 Timisoara, Romania; vaduva.adrian@umft.ro; 10 Department of Pathology, “Pius Brinzeu” County Clinical Emergency Hospital, 300723 Timisoara, Romania; 11 Burn Unit, Plastic and Reconstructive Surgery Department Casa Austria, County Emergency Clinical Hospital “Pius Branzeu”, 300723 Timisoara, Romania; 12 Plastic Surgery Department, Victor Babes University of Medicine and Pharmacy, 300041 Timisoara, Romania; crainiceanu.zorin@umft.ro; 13 Department XV, Discipline of Urology, Victor Babes University of Medicine and Pharmacy Timisoara, E. Murgu Square, No. 2, 300041 Timisoara, Romania; razvan.bardan@umft.ro

**Keywords:** Integra^®^, dermal matrix, soft tissue defects, lymphoma, chemotherapy

## Abstract

Background/Objectives: Subcutaneous panniculitis-like T-cell lymphoma (SPTCL) is a rare and aggressive cutaneous lymphoma, often misdiagnosed due to nonspecific clinical features. Early diagnosis and treatment remain challenging. Methods: We report the case of a 31-year-old female with a chronic non-healing gluteal wound initially treated as an abscess. The lack of improvement prompted repeated investigations, culminating in the diagnosis of SPTCL with an alpha–beta T-cell phenotype. Results: Management involved combined chemotherapy and surgical wound reconstruction. Six cycles of CHOEP-21 chemotherapy led to complete clinical remission. A soft tissue defect superinfected with multidrug-resistant organisms was successfully reconstructed using Integra Dermal Regeneration Template followed by split-thickness skin grafting. Conclusions: This case highlights the diagnostic complexity of SPTCL and the therapeutic potential of dermal matrix application in complex wound management, especially in immuno-compromised patients.

## 1. Introduction

Deep skin infections, including abscesses, can have an unpredictable evolution, especially in the presence of underlying hematologic disorders. Subcutaneous panniculitis-like T-cell lymphoma (SPTCL) is a rare, extranodal variant of peripheral T-cell lymphoma that primarily affects the subcutaneous adipose tissue, often mimicking benign inflammatory conditions such as panniculitis, cellulitis, or autoimmune panniculitis disorders [1,2,3]. Its rarity, nonspecific clinical presentation, and resemblance to more common cutaneous or infectious pathologies contribute to frequent misdiagnosis and the delayed initiation of appropriate therapy [1,4]. SPTCL typically presents as multiple subcutaneous nodules or plaques, predominantly involving the extremities and trunk. These are often associated with systemic symptoms such as fever, fatigue, and weight loss. Histopathologically, the disease is characterized by lobular panniculitis with atypical T-cell infiltrates, the rimming of adipocytes by pleomorphic lymphocytes, the presence of histiocytes and karyorrhexis, and the absence of epidermal or dermal involvement [5,6,7]. SPTCL is classified into alpha/beta (α/β) and gamma/delta (γ/δ) subtypes based on T-cell receptor phenotype [2,8]. The α/β subtype (CD3+, CD8+, TIA-1+, granzyme B+) has a favorable prognosis, while the γ/δ subtype (CD56+, CD4–, CD8–) is associated with hemophagocytic syndrome and worse outcomes [2,6,9,10,11]. The World Health Organization—European Organization for Research and Treatment of Cancer (WHO-EORTC) now classifies only α/β cases as SPTCL, reclassifying γ/δ forms as PCGD-TCL [2]. The diagnosis of SPTCL requires a high index of suspicion and relies on deep incisional or excisional biopsy, histopathologic evaluation, and immunophenotyping. Imaging such as PET/CT may assist in staging but is often non-specific in early disease [4,7]. The average time from symptom onset to definitive diagnosis can exceed two years, largely due to the disease’s ability to masquerade as infectious or inflammatory panniculitis [12]. Therapeutic strategies vary based on subtype and clinical severity. While SPTCL-AB may respond to immunosuppressive therapy (e.g., corticosteroids, cyclosporine), aggressive disease or the presence of hemophagocytic syndrome often necessitates combination chemotherapy regimens such as CHOP (Cyclophosphamide, Doxorubicin, Vincristine, Dexamethasone) or CHOEP (Cyclophosphamide, Doxorubicin, Vincristine, Etoposide, Dexamethasone) [13,14,15,16]. Nonetheless, the optimal treatment remains controversial due to the rarity of the disease and limited prospective data. We present the case of a young female with a delayed diagnosis of SPTCL, initially diagnosed and managed as a gluteal abscess. In addition to detailing the diagnostic and therapeutic process, we highlight a novel reconstructive strategy involving the application of Integra^®^ Dermal Regeneration Template, a synthetic dermal substitute traditionally used in burn and traumatic wounds, in the context of an immunocompromised oncologic patient.

## 2. Materials and Methods

**Figure 1:**
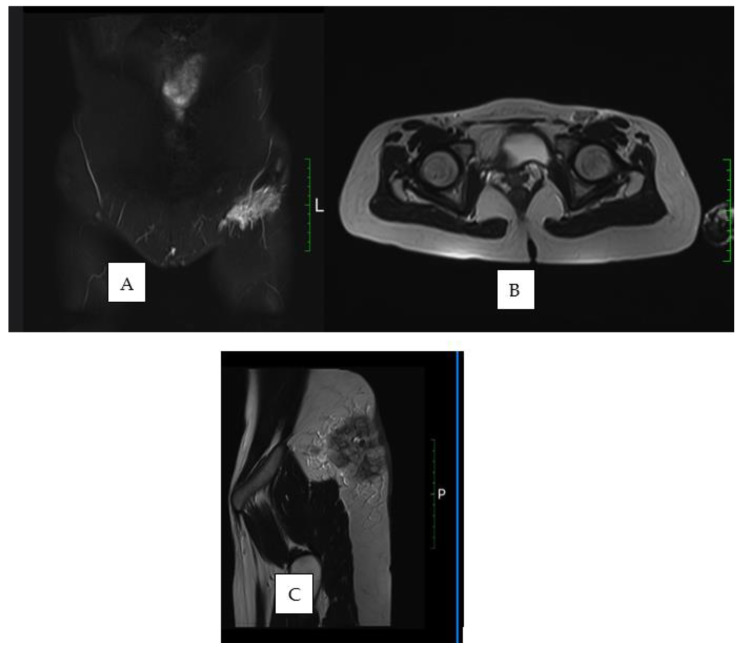
(**A**) Axial CT view showing left inguinal adenopathy; (**B**) Sagittal CT view revealing edematous and infiltrative changes in the left inguinal region; (**C**) Sagittal CT view of the gluteal region demonstrating a skin defect and profound subcutaneous edema.

We present the case of a 31-year-old female patient who was initially admitted to a territorial general surgery unit for a painful, inflammatory swelling in the left gluteal region associated with persistent fever exceeding 38 °C. The initial presumptive diagnosis was a gluteal abscess, for which incision and drainage were performed, and a tissue biopsy sample was collected for histopathological analysis. Empirical antibiotic therapy was initiated. However, due to a lack of clinical improvement, the patient was referred to a regional surgical center, where she was managed for what was presumed to be a recurrent gluteal abscess. The incision was extended, followed by local wound care and continued antibiotic treatment. Despite these interventions, the wound failed to heal and progressed into an atonic wound with cutaneous and soft tissue loss. The persistence of febrile episodes and a deteriorating general condition led to her transfer to the Infectious Diseases Department for further evaluation. During hospitalization, a comprehensive workup was performed, including magnetic resonance imaging and thoraco-abdomino-pelvic computed tomography. Imaging revealed diffuse inflammatory changes in the subcutaneous tissue of the left gluteal region, without involvement of deeper structures. In addition, multiple lymphadenopathies were identified in the left inguinal and external iliac regions (Figure 1).

**Figure 2:**
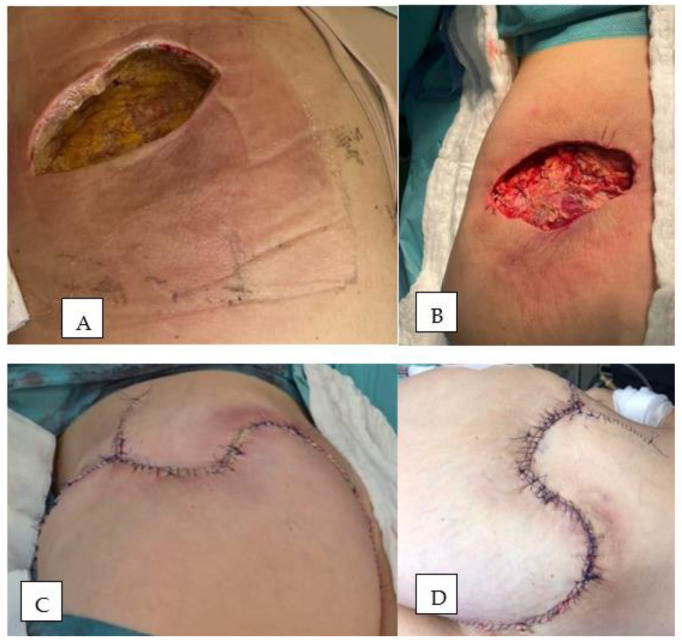
(**A**) Initial appearance of the wound at referral; (**B**) Intraoperative image during extensive debridement; (**C**) Intraoperative view showing defect coverage using two rotational flaps; (**D**) Postoperative appearance on day 2.

Infectious disease screening panels were inconclusive, and initial histopathological findings suggested only a non-specific inflammatory process. The patient received broad-spectrum antibiotics, corticosteroids, and supportive care, with no significant clinical response. On 26 July 2024, due to persistent infection with Proteus mirabilis and the presence of a soft tissue defect measuring approximately 8 × 5 cm (Figure 2A), the patient was transferred to the Plastic Surgery Department for specialized surgical management. She underwent extensive debridement of necrotic tissue (Figure 2B), wound lavage, repeat tissue sampling for histology and culture, and the initiation of negative pressure wound therapy (NPWT). The histological analysis again revealed adipose tissue with discrete fibrotic septa and widespread necrobiosis accompanied by acute fibrino-granulocytic inflammation. With local stabilization and sterile culture results, reconstruction was performed using two rotational cutaneous flaps in August 2024 (Figure 2C,D).

**Figure 3:**
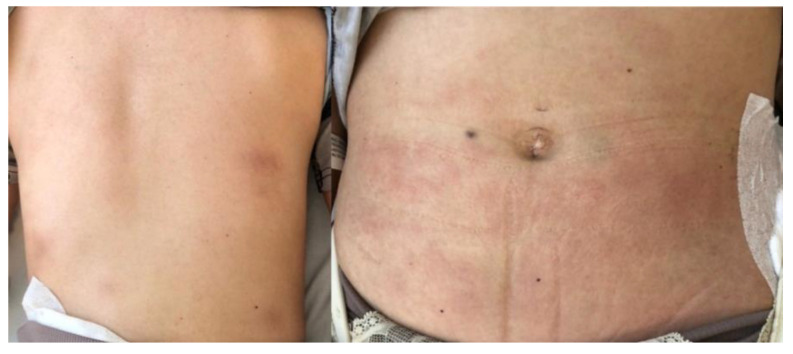
Inflammatory subcutaneous nodules located on the posterior trunk and lower abdominal wall.

**Figure 4:**
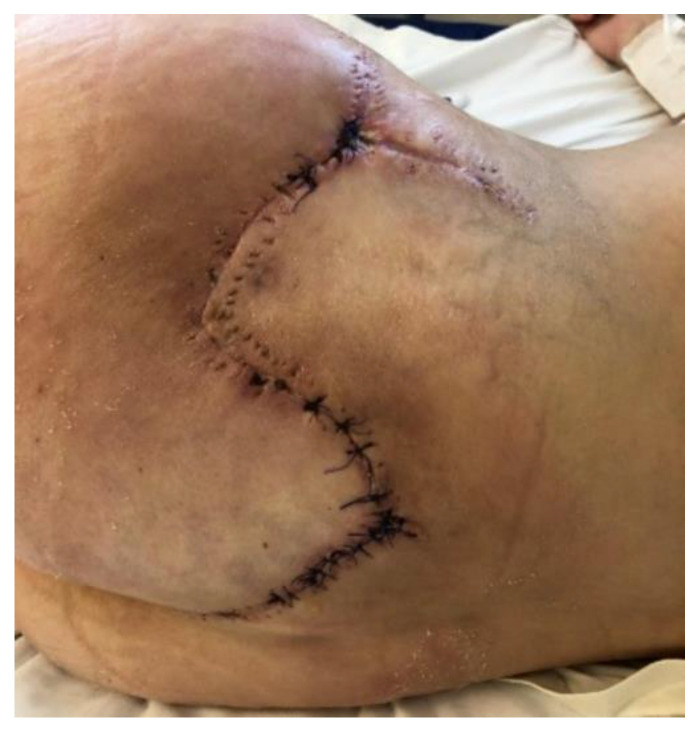
Local wound examination at readmission showing minimal dehiscence.

Three weeks after surgery, the patient developed multiple painful subcutaneous nodules on the posterior thorax and lower abdomen (Figure 3), along with progressive inguinal lymphadenopathy and recurrent fever over 38 °C. Her general condition deteriorated, necessitating hospital readmission. An examination of the gluteal wound revealed a small dehiscent area with mild discharge (Figure 4).

**Figure 5:**
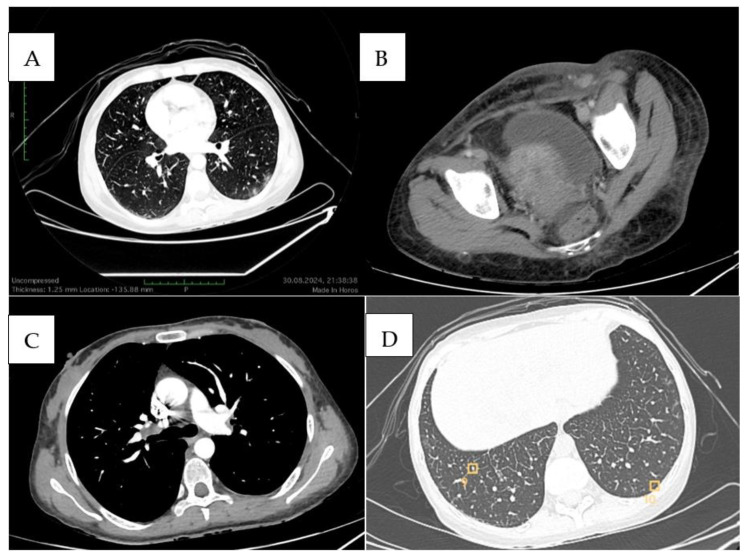
(**A**) Pulmonary window with left subpleural alveolar lesions; (**B**) Left inguinal adenopathy, intensely capturing the contrast substance and impingement phenomena in subcutaneous cellular tissue; (**C**) Mediastinal window with right hilar adenopathy; (**D**) Pulmonary window with lung micronodules.

A new thoraco-abdomino-pelvic CT scan performed on 30 August 2024 revealed interstitial pulmonary changes, a 3 mm subpleural pulmonary nodule, and bilateral inguinal adenopathy, with significant enhancement on the left, associated with subcutaneous plane impingement. Diffuse infiltrative edema in the left gluteal region associated with significant soft tissue changes over an 18 × 15 cm area was also noted (Figure 5).

**Figure 6:**
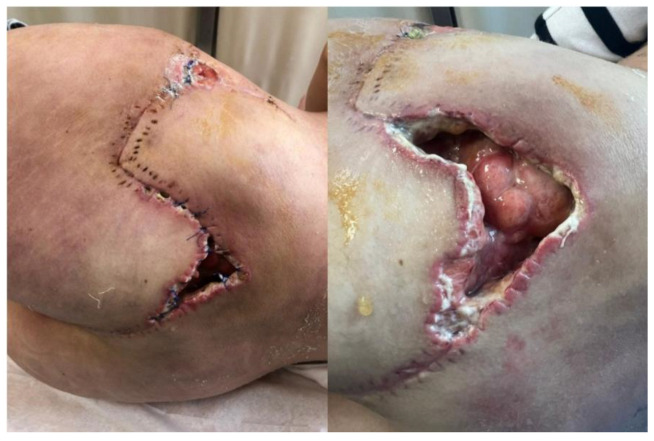
Wound infection with Pseudomonas and progression of dehiscence.

Following interdisciplinary consultations during the hospitalization, the suspicion of an autoimmune disease, specifically sarcoidosis associated with panniculitis nodosa (Weber–Christian disease), was raised. Still, all the specific tests recommended were negative, and only non-specific changes were observed. Immunosuppressive treatment with mycophenolate mofetil, plaquenil, and prednisone was initiated. However, the local evolution was unfavorable, and the wound became dehiscent and infected with multidrug-resistant *Pseudomonas aeruginosa*, resulting in an atonic wound with cutaneous and soft tissue defects, requiring further debridement (Figure 6).

**Figure 7:**
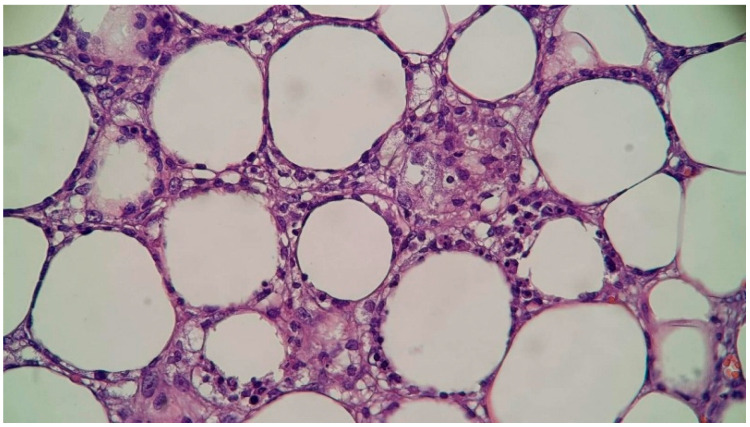
High-power view of adipocytic rimming of lymphocytes, along with some histiocytes and neutrophils (40× magnification).

**Figure 8:**
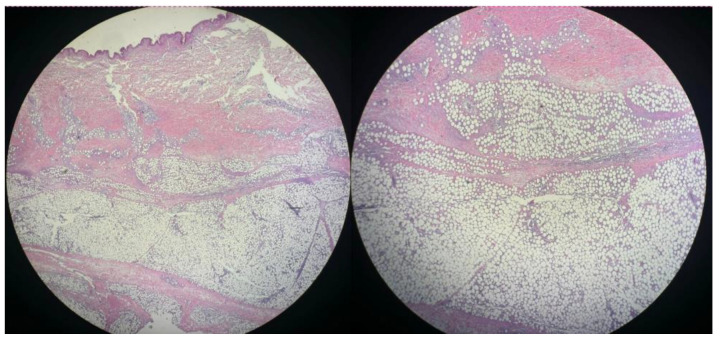
Lower magnification shows a dense inflammatory infiltrate in the dermis and subcutaneous fat tissue (**left**, 2× magnification); Higher magnification of the previous image reveals a dense inflammatory infiltrate primarily within the adipocytic lobules with reduced interlobular septal involvement (**right**, 2× magnification).

**Figure 9:**
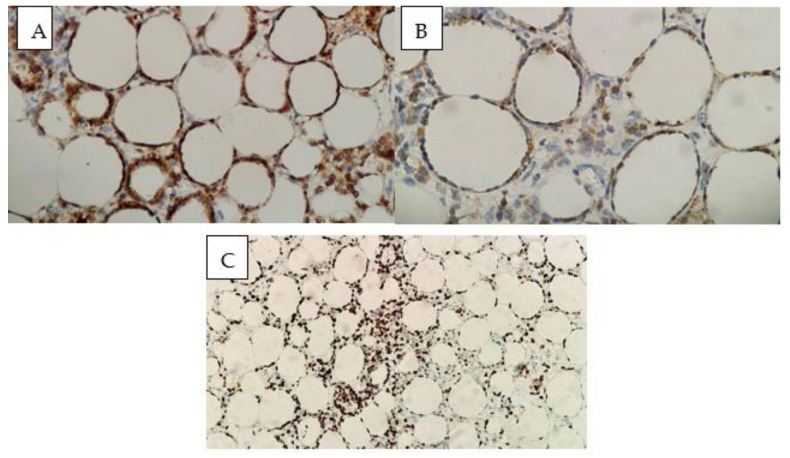
Immunohistochemistry demonstrates (**A**) Medium-sized T-cell infiltrate with diffuse CD3 positivity (40× magnification); (**B**) Loss of CD5 expression (40× magnification); (**C**) High proliferative index (Ki-67: 60–65%, 20× magnification)).

In consideration of the persistent symptoms and the lack of concrete diagnosis, a subcutaneous nodule was excised from the posterior thoracic cavity and sent for histopathologic examination. In this context, the suspicion of cutaneous tuberculosis was also raised, so the patient was briefly admitted in the Pneumology Department, where bronchoscopy with bronchoalveolar lavage and bronchial aspirate were performed, but the tests for TB were negative. Histological examination revealed a predominant lymphocytic infiltrate involving the adipocytic lobules that minimally extended into the interlobular septa. The lymphocytes showed some rimming of the adipocytes, with a few histiocytes and neutrophils accompanying them. Apoptotic figures and necrosis were noted. These features were suggestive of subcutaneous panniculitis-like T-cell lymphoma (SPTCL) (Figure 7 and Figure 8). Immunohistochemistry showed a medium-sized T-cell infiltrate with diffuse CD3 positivity, a loss of CD5 expression, and a high proliferative index (Ki-67: 60–65%) (Figure 9). Additional stains showed the following profile: Granzyme B- and CD8-positive, CD4- and CD56-negative. The immunophenotype and morphologic aspects favored the diagnosis of peripheral T-cell lymphoma with α/β T-cell receptor phenotype.

Following diagnosis, the patient was referred to a Hematology Department (at the Fundeni Clinical Institute in Bucharest). Due to the complexity of the wound and ongoing multidrug-resistant infection, immediate chemotherapy initiation was deferred. Subsequently, upon arrival at our clinic, a multidisciplinary treatment plan was agreed in collaboration with the local Hematology Department, involving systemic chemotherapy and the continued surgical management of the gluteal wound.

### Therapeutic Management: Systemic and Surgical Wound Treatment

After obtaining the patient’s informed consent and following a multidisciplinary consensus, specific CHOEP-21 chemotherapy was initiated, in parallel with surgical wound care. The specific chemotherapy protocol consisted of six cycles of treatment, given at 21-day intervals:

- Cyclophosphamide—1100 mg (Day 1);
- Doxorubicin—75 mg (Day 1);
- Vincristine—2 mg (Day 1);
- Etoposide—150 mg (Days 1, 3, 5);
- Dexamethasone—16 mg (Days 1–5).

**Figure 10:**
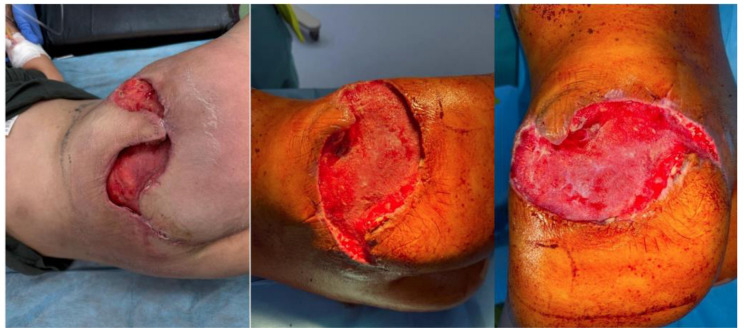
Serial debridement and NPWT between chemotherapy sessions.

Following the first cycle, surgical debridement and NPWT dressing change were performed. The patient developed febrile neutropenia (leukocyte count 200/mm^3^), necessitating granulocyte growth factor (G-CSF) therapy and broad-spectrum antimicrobial and antifungal prophylaxis. Clinical response became evident during the second cycle, with a regression of subcutaneous nodules and lymphadenopathy. Between cycles, the patient underwent regular wound debridement, NPWT maintenance, and microbiological surveillance of the Pseudomonas infection. Granulation tissue formation improved steadily (Figure 10).

**Figure 11:**
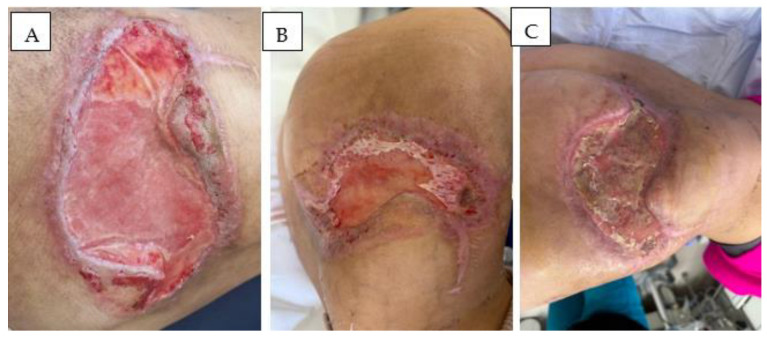
(**A**) Integra: 3 weeks post-application (>90% integrated); (**B**) STSG at 3 weeks post-application (75% lysed); (**C**) Granulation tissue prior to Hyalo4 Regen sponge application.

Between the third and fourth chemotherapy cycles, the wound defect was covered with a double-layer Integra^®^ Dermal Regeneration Template (Integra LifeSciences, Plainsboro, NJ, USA), over which the NPWT dressing was maintained. Despite chemotherapy-induced immunosuppression, matrix integration exceeded 90% (Figure 11A). Three weeks later, a definitive coverage of the defect was achieved with a split-thickness skin graft (STSG) applied, followed by further NPWT. After the fifth cycle, the dressing was discontinued, though approximately 75% graft lysis was observed (Figure 11B). Viable granulation tissue persisted, forming an appropriate substrate for re-epithelialization (Figure 11C).

**Figure 12:**
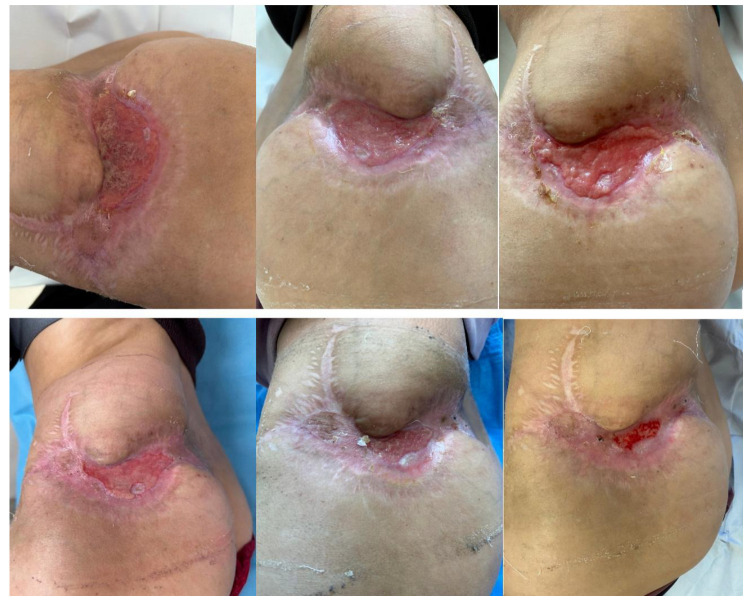
Progressive wound healing with Hyalo4 Regen sponge application.

To promote epithelialization, Hyalo4 Regen sponge dressings were applied every 3–4 days. The skin defect area gradually reduced and re-epithelialized (Figure 12). After completing six chemotherapy cycles, the patient achieved full clinical remission, with the disappearance of nodules and the resolution of systemic symptoms.

**Figure 13:**
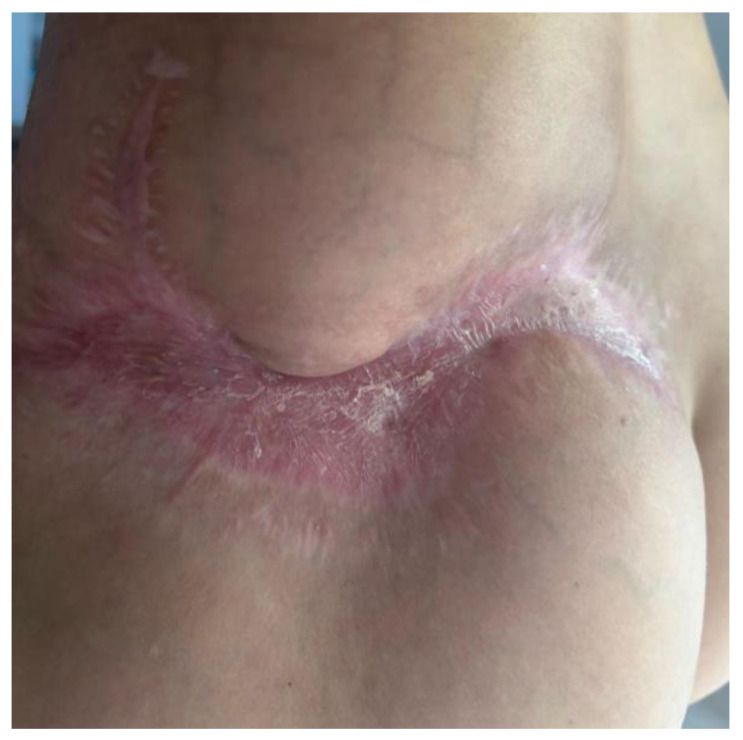
Complete healing of the wound.

One month after the end of treatment, the complete healing of the left gluteal defect was observed, with no signs of local or systemic recurrence (Figure 13).

## 3. Discussion and Literature Review

### 3.1. Epidemiology

Subcutaneous panniculitis-like T-cell lymphoma (SPTCL) is a rare form of non-Hodgkin lymphoma (NHL) characterized by the infiltration of the subcutaneous adipose tissue by neoplastic cytotoxic T-lymphocytes, without the involvement of the dermis or epidermis. While NHL represents 90% of all lymphomas and arises from either B- or T-cell lineages, SPTCL is a rare primary cutaneous T-cell lymphoma comprising fewer than 1% of all NHLs [3]. The median age of onset is approximately 36 years, with a slight female predominance. The pathogenesis of SPTCL remains incompletely understood but is likely multifactorial, involving genetic predisposition, chronic antigenic stimulation, immune dysregulation, and associations with autoimmune conditions or viral infections [2].

### 3.2. Histopathology

Histopathologically, SPTCL is defined by lobular panniculitis with a neoplastic infiltrate composed predominantly of pleomorphic cytotoxic T-cells that surround and destroy adipocytes (“rimming”), often accompanied by histiocytes, karyorrhexis, and variable degrees of necrosis [5]. The dermis and epidermis are typically spared. In many cases, associated systemic features include fever, weight loss, hepatosplenomegaly, cytopenias, and, in more aggressive cases, hemophagocytic lymphohistiocytosis (HLH) [6].

Diagnosing cutaneous T-cell lymphoma (CTCL) through histopathology and immunohistochemistry poses significant challenges, as outlined in the recent literature, including the updates provided by the European Organisation for Research and Treatment of Cancer (EORTC). CTCL is characterized by a variety of clinical and histologic presentations, often mimicking benign inflammatory dermatoses, which complicates the diagnostic process [17,18,19].

Histologically, common diagnostic features in CTCL include a predominance of CD4+ T-cells, atypical lymphocyte forms known as cerebriform nuclei, and the presence of epidermotropism and Pautrier microabscesses. However, subtle variations in presentation can result in misdiagnosis [17,19]. For instance, early Mycosis Fungoides (MF), the most prevalent subtype of CTCL, frequently presents similarly to atopic dermatitis, leading to diagnostic delays averaging six years [20,21]. This diagnostic ambiguity is further compounded by overlapping features with other dermatological conditions such as drug reactions and lymphomatoid dermatitis [22].

Immunohistochemistry plays a crucial role in distinguishing cutaneous T-cell lymphoma (CTCL) from other skin malignancies and benign entities. Key immunophenotypic markers include the loss of pan-T-cell antigens (like CD2, CD3, and CD5), although these markers can be variably expressed in both inflammatory and neoplastic conditions, leading to potential misinterpretations [17,23]. Furthermore, the role of molecular analysis in assessing T-cell receptor (TCR) clonality via advanced techniques, such as next-generation sequencing, can provide definitive evidence of malignancy when histopathological features are inconclusive. This approach has shown a significant correlation between clonality findings and diagnostic confirmations under challenging cases [18,24].

Also pertinent to the discussion of diagnostic challenges is the background of therapeutic interventions, such as biologics like dupilumab, which may alter the immune environment and clinical presentation, ostensibly masking underlying malignancies like CTCL [25]. Thus, a careful consideration of patients presenting with dermatitis who have undergone treatments that modulate the immune response is critical to avoid misdiagnosis and inappropriate therapies [26,27].

To further complicate matters, there remains a lack of standardization in the diagnostic criteria among dermatopathologists. Variability in the approach to histopathologic examination necessitates consultation with an experienced dermatopathologist to navigate these challenges effectively [28].

### 3.3. Clinical Manifestation and Evaluation

The clinical presentation of SPTCL is heterogeneous but usually involves multiple painless subcutaneous nodules or plaques predominantly located on the trunk and extremities. These may be purpuric or ulcerated and, in some patients, evolve over time in morphology and distribution. Delayed diagnosis is common, as initial lesions may resemble panniculitis, abscesses, or autoimmune panniculitis conditions [4].

The classification of SPTCL has evolved based on immunophenotypic markers and T-cell receptor (TCR) subtypes. The WHO-EORTC classification distinguishes two major subtypes: SPTCL with α/β TCR expression (SPTCL-AB)—usually CD3+, CD8+, TIA-1+, granzyme B+, CD4−, CD30−, and CD56− (this subtype has an indolent clinical course and favorable prognosis); and SPTCL with γ/δ TCR expression (SPTCL-GD)—characterized by CD4−, CD8−, frequent CD56 co-expression, and an aggressive course, often with associated HLH [6,7,8,9,10,11].

Due to differences in clinical behavior and prognosis, the WHO-EORTC classification currently reserves the term “SPTCL” for the α/β subtype, whereas the γ/δ variant is categorized separately as primary cutaneous γ/δ T-cell lymphoma (PCGD-TCL), a provisional entity within peripheral T-cell lymphomas, not otherwise specified [8].

Despite this classification, the literature remains inconsistent, and many reported cases do not provide complete immunophenotyping, complicating subgroup analyses. Further validation in larger cohorts is needed to solidify the prognostic and therapeutic implications of this subdivision [10].

Diagnostic evaluation involves deep skin biopsy, preferably incisional or excisional, targeting subcutaneous nodules. Immunohistochemistry is critical for confirming T-cell lineage and cytotoxic phenotype. Common markers include CD3, CD8, TIA-1, granzyme B, and βF1 (TCR-α/β), with negative staining for CD4 and CD56 in most SPTCL-AB cases [5,9].

Advanced imaging, particularly PET/CT, supports staging and treatment monitoring by identifying metabolically active lesions and systemic involvement [29]. Although not specific for diagnosis, PET/CT can reveal the true extent of disease and assist in evaluating therapeutic response [16].

Differential diagnosis is extensive due to the overlapping features with inflammatory, infectious, and neoplastic panniculitides. Key differentials include the following. Lupus profundus (lupus panniculitis): Characterized by lymphocytic and plasmacytic infiltrates with fibrinoid necrosis in perivascular connective tissue; both CD4+ and CD8+ T-cells are typically present, and CD123+ plasmacytoid dendritic cells are prominent; lacks cytologic atypia and clonality [7]. Primary cutaneous γ/δ T-cell lymphoma (PCGD-TCL): Involves epidermis and dermis with ulcerated plaques and epidermotropism; immunophenotype includes CD56 and TCR-γ positivity, with aggressive progression [4,7]. Extranodal NK/T-cell lymphoma, nasal type: Characterized by EBV positivity, CD56+, CD8−, extensive angioinvasion, and dermal/epidermal involvement; not limited to subcutaneous tissue and often involves the upper aerodigestive tract [4]. Anaplastic large cell lymphoma (ALCL): Often presents with epidermal ulceration and strong CD30 expression; may mimic nodular panniculitis clinically [7].

SPTCL diagnosis is frequently delayed due to its rarity and overlapping presentation with benign panniculitides. One systematic review found that 20% of analyzed cases were initially misdiagnosed [10,11,29]. Thus, early biopsy with extended immunohistochemistry and correlation with imaging and clinical context is crucial.

Overall, SPTCL-AB has a favorable prognosis, with a 5-year survival rate exceeding 80% in the absence of HLH [15,16]. Disease course is often chronic and relapsing, though the spontaneous regression of lesions has been described [13]. HLH is characterized by prolonged fever, hepatosplenomegaly, pancytopenia, hyperferritinemia, and the evidence of hemophagocytosis in bone marrow or tissue biopsies and occurs in a subset of patients associated with elevated mortality; thus, its identification is essential in risk stratification and therapeutic planning [30,31].

### 3.4. Treatment Options

The treatment of SPTCL remains non-standardized due to the rarity of the disease and its variable clinical behavior. Available therapies include systemic corticosteroids, immunosuppressive agents such as cyclosporine, and multi-agent chemotherapy regimens. The choice of treatment is often guided by clinical aggressiveness, immunophenotype, and the presence of complications such as hemophagocytic syndrome (HLH) [30,31].

Given the absence of universally accepted guidelines, therapeutic approaches have historically mirrored those used in other cutaneous T-cell lymphomas. Corticosteroids are often employed as first-line therapy, particularly in indolent cases. In a retrospective analysis by Go and Wester, 20 patients received corticosteroids as initial therapy; 30% achieved complete remission and 20% partial remission. However, relapses occurred in most patients within six months of corticosteroid withdrawal, often requiring salvage chemotherapy or resulting in disease-related mortality [13].

Immunosuppressive agents, particularly cyclosporine, have been investigated as steroid-sparing options. Cyclosporine inhibits T-cell activation by downregulating interleukin-2 and other pro-inflammatory cytokines involved in T-cell proliferation [32]. Shani-Adir et al. first reported the successful use of cyclosporine in two pediatric patients with SPTCL, both of whom achieved initial remission; one later relapsed and required chemotherapy [14]. Rojnuckarin et al. extended these findings to adults, reporting on four patients with relapsed or refractory SPTCL following CHOP-based chemotherapy. All responded rapidly to cyclosporine (4 mg/kg/day), with three achieving durable complete remission [15].

The aggressiveness of the lymphocytic infiltrate often determines whether immunosuppressants alone are sufficient. In patients with a more indolent course, monotherapy with prednisone, methotrexate, or cyclophosphamide may be adequate. For aggressive cases or those unresponsive to immunosuppressants, anthracycline-based chemotherapy regimens such as CHOP or CHOEP remain the mainstay [4,6]. Cyclosporine, alone or combined with corticosteroids, has also demonstrated efficacy in cases complicated by HLH [15,31].

Before the immunophenotypic distinction between α/β and γ/δ TCR subtypes, most SPTCL patients were treated as if they had aggressive disease. Consequently, doxorubicin-based chemotherapy was widely adopted, sometimes combined with monoclonal antibodies (e.g., alemtuzumab) or followed by autologous stem cell transplantation (auto-SCT) [6,33,34]. However, an increasing recognition of the indolent behavior of SPTCL-AB has prompted a reevaluation of this approach.

Vitamin D deficiency has been correlated with adverse outcomes in patients diagnosed with lymphoid malignancies. Recent evidence indicates that diminished levels of vitamin D at the time of diagnosis may be associated with decreased survival rates and an elevated risk of disease progression. Furthermore, vitamin D supplementation seems to improve tumor response to treatment, especially in patients undergoing immunochemotherapy. These findings underscore the significance of routinely evaluating vitamin D levels in all lymphoma patients as a component of the initial assessment and continuous management [35].

Evidence from retrospective studies supports a more conservative strategy in SPTCL-AB patients without HLH. Willemze et al. reported that, among 52 SPTCL-AB patients without HLH, only two deaths occurred—one due to treatment toxicity and one from unrelated causes [1]. In their cohort, 64% of patients treated with CHOP-like regimens achieved sustained complete remission. Similarly, immunosuppressive agents achieved sustained complete remission in 55% of patients, with some responding after re-treatment [1].

In addition to systemic therapy, localized disease may be managed with radiotherapy or surgical excision. Complete remission following local treatments was reported in five patients with solitary or limited lesions. These findings suggest that a universal, aggressive therapeutic approach is not necessary for all patients, especially those with low-risk features [1].

The management of integumentary and subcutaneous tissue defects can be particularly challenging, especially in cases complicated by infection, extensive tissue necrosis, or after oncologic surgical excision. These scenarios require effective reconstructive strategies that ensure wound coverage, prevent secondary infection, and support dermal regeneration, particularly in immunocompromised patients undergoing chemotherapy.

Integra^®^ Dermal Regeneration Template (IDRT; Integra LifeSciences, Princeton, NJ, USA) is among the most advanced solutions available for dermal reconstruction. Originally designed for the management of full-thickness burns, its clinical use has expanded into trauma, tumor resection, chronic wounds, and reconstructive procedures in compromised patients [36,37]. IDRT consists of a bi-layered acellular matrix composed of a porous dermal analog made from cross-linked type I bovine collagen and shark-derived chondroitin-6-sulfate, overlaid by a semi-permeable silicone layer that acts as a temporary epidermal substitute. The collagen scaffold is cross-linked using glutaraldehyde to enhance durability and mechanical strength [38].

The regeneration process with Integra^®^ follows four coordinated biological stages: (1) imbibition (early protein absorption), (2) fibroblast migration into the matrix, (3) neovascularization, and (4) remodeling and maturation into neodermis [39]. The silicone layer prevents dehydration and acts as a bacterial barrier, which is particularly advantageous in wounds colonized or infected with multidrug-resistant organisms. In SPTCL patients with immunosuppression and chronic wounds, such protection is vital for successful healing.

Although Integra^®^ was originally approved for burn treatment, multiple studies have validated its efficacy in reconstructing complex wounds of the trunk, extremities, and head and neck areas, particularly when autologous skin grafting is not feasible or is delayed [38,40]. Its application in the oncologic setting—including hematological malignancies with cutaneous involvement—has been reported but remains rare. In SPTCL, where recurrent or aggressive lesions often require serial surgical interventions, Integra^®^ provides a versatile, staged approach that supports early dermal regeneration while deferring definitive epidermal closure.

Published data on the use of Integra^®^ in patients with cutaneous lymphoma are extremely limited. To date, only two cases have been described. In one, Integra^®^ was used successfully to reconstruct a full-thickness burn wound in a patient with cutaneous lymphoma [41]. In another, it was applied to cover a post-excisional defect following surgical treatment of a primary peripheral cutaneous T-cell lymphoma [42].

Despite this limited experience, the rationale for using Integra^®^ in SPTCL is strong. The matrix provides an effective bridge to definitive wound closure in complex clinical settings marked by immunosuppression, cytotoxic treatment, and colonization with resistant pathogens. Its role in reducing the need for more invasive flap procedures is particularly relevant when local tissues are compromised or when systemic factors contraindicate prolonged surgery.

Most studies on subcutaneous panniculitis-like T-cell lymphoma (SPTCL) are retrospective or based on isolated case reports. Due to the disease’s rarity and diverse clinical presentations, case-based evaluations remain crucial to better understand the variable evolution and optimize diagnostic strategies [12].

Our patient exemplifies the challenges inherent to diagnosing and managing SPTCL. The disease initially presented as a chronic, painful, and non-healing gluteal lesion, misinterpreted as an abscess and treated empirically. Despite repeated surgical drainage and broad-spectrum antibiotics, the condition worsened. A histopathologic evaluation of two successive gluteal biopsies failed to yield a definitive diagnosis, reflecting the known difficulty of identifying SPTCL in its early stages. The diagnosis was finally established after a third biopsy—this time from a distant subcutaneous nodule on the posterior thorax—which revealed characteristic histological features and immunophenotypic markers of SPTCL with α/β T-cell receptor subtype. This delay of approximately 2.5 months from initial presentation to diagnosis, although shorter than the average reported in the literature, illustrates the disease’s capacity to mimic inflammatory, infectious, or autoimmune panniculitides [3,6,12].

The patient’s presentation lacked hepatosplenomegaly, and no histological signs of hemophagocytosis were observed, effectively excluding associated hemophagocytic lymphohistiocytosis (HLH), a known negative prognostic factor. Following diagnosis, CHOEP-21 chemotherapy was initiated despite the presence of a multidrug-resistant Pseudomonas aeruginosa wound infection in the gluteal region. Systemic therapy was well-tolerated, and, by the completion of the six-cycle regimen, the patient achieved a complete clinical response, including the resolution of systemic symptoms, regression of inguinal lymphadenopathy, and disappearance of subcutaneous nodules.

However, the local management of the gluteal wound represented an additional challenge. After initial surgical debridement and flap failure, Integra^®^ Dermal Regeneration Template was applied as part of a combined reconstructive–oncologic approach. Despite concurrent chemotherapy and systemic immunosuppression, the dermal matrix demonstrated > 90% integration. This was followed by split-thickness skin grafting and additional conservative measures (e.g., Hyalo4 dressings), ultimately resulting in complete wound healing without recurrence.

To the best of our knowledge, this is the first published case of Integra^®^ being successfully applied in an actively immunosuppressed patient with SPTCL undergoing anthracycline-based chemotherapy. Our experience therefore expands the current understanding of Integra^®^’s role in complex clinical settings, particularly where reconstructive options are limited due to infection risk, tissue fragility, or ongoing cytotoxic therapy.

## 4. Conclusions

Subcutaneous panniculitis-like T-cell lymphoma (SPTCL) is a rare and diagnostically challenging cutaneous lymphoma that frequently mimics benign inflammatory conditions. Due to its nonspecific presentation and histological overlap with panniculitis, early diagnosis requires a high index of suspicion, prompt biopsy of nodular lesions, and immunophenotyping using markers such as CD2, CD3, CD8, granzyme B, and TIA-1.

This case highlights the importance of a multidisciplinary approach involving hematology, plastic surgery, infectious diseases, and dermatology for timely diagnosis and coordinated management. Despite an initial delay in diagnosis, the patient achieved complete remission of systemic disease following six cycles of CHOEP-21 chemotherapy. In parallel, adjuvant surgical reconstruction of a superinfected gluteal defect was successfully achieved using Integra^®^ Dermal Regeneration Template.

Integra^®^ proved to be a viable and innovative option for managing extensive tissue loss in an immunocompromised oncologic setting, offering effective neodermal regeneration and reducing the need for more invasive flap procedures. Although the literature reports on its use in hematologic malignancies remain limited, our experience suggests that Integra^®^ may be particularly beneficial in complex wounds where autologous grafting is not feasible.

This case underscores the value of early tissue sampling, timely access to advanced diagnostics, and personalized, multidisciplinary therapeutic strategies. Future prospective studies are needed to validate the broader use of dermal substitutes like Integra^®^ in cutaneous lymphomas and to develop standardized protocols for their integration into oncologic wound care.

## Data Availability

The original contributions presented in the study are included in the article. Further inquiries can be directed to the corresponding author.
